# Supplementary figures and images for: Identification of the biological function of miR-9 in spinal cord ischemia-reperfusion injury in rats
Source: PeerJ. 2021 May 13;9:e11440. doi: 10.7717/peerj.11440 (PMC8126262; doi:10.7717/peerj.11440)

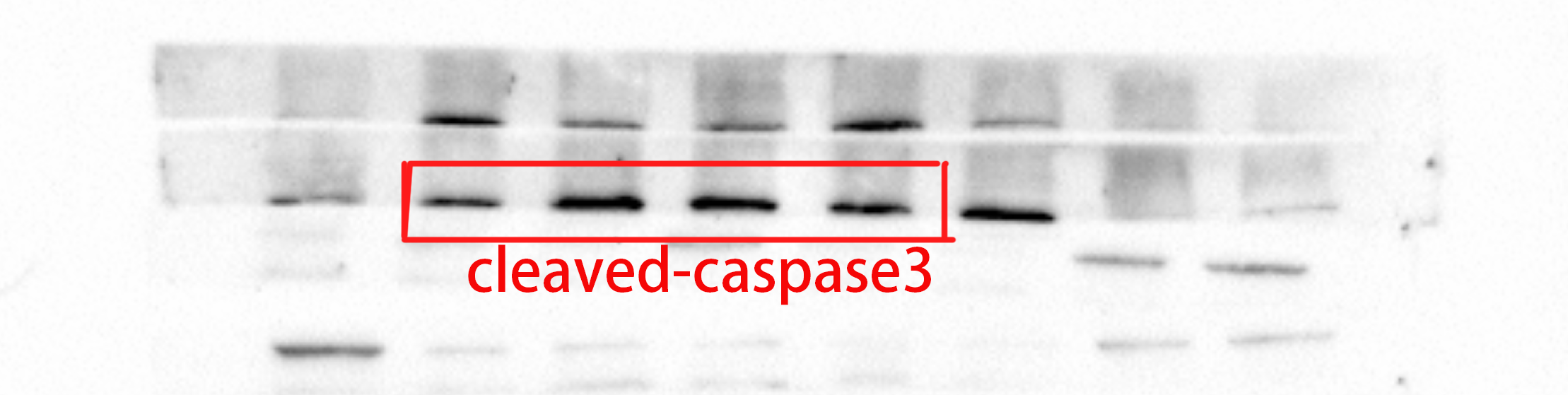

Supplement: Supplemental Information 3 [file peerj-09-11440-s003.zip › Uncropped GelsBlots/Figure4C.tif]

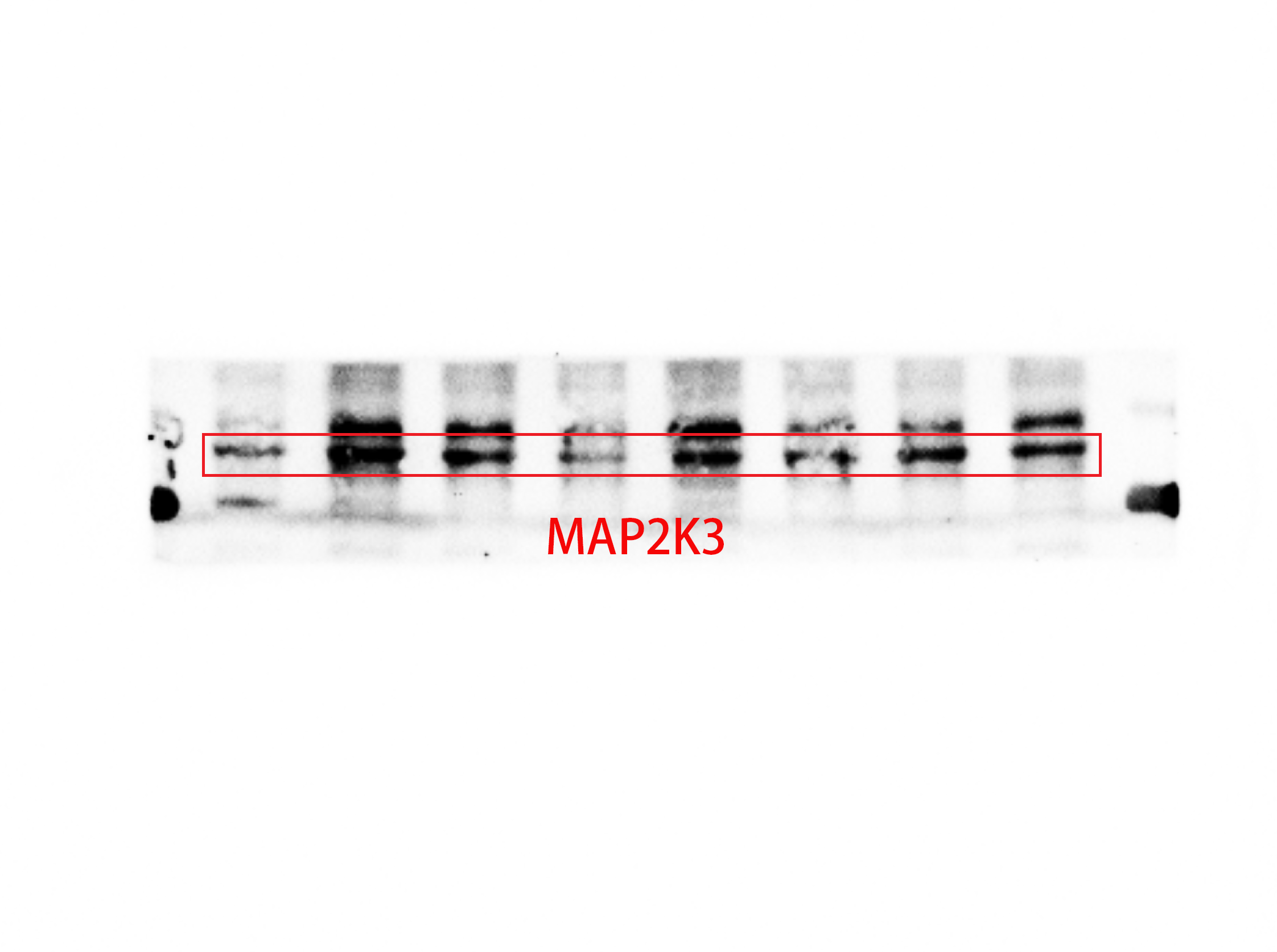

Supplement: Supplemental Information 3 [file peerj-09-11440-s003.zip › Uncropped GelsBlots/Figure6B.MAP2K3╘¡═╝.tif]

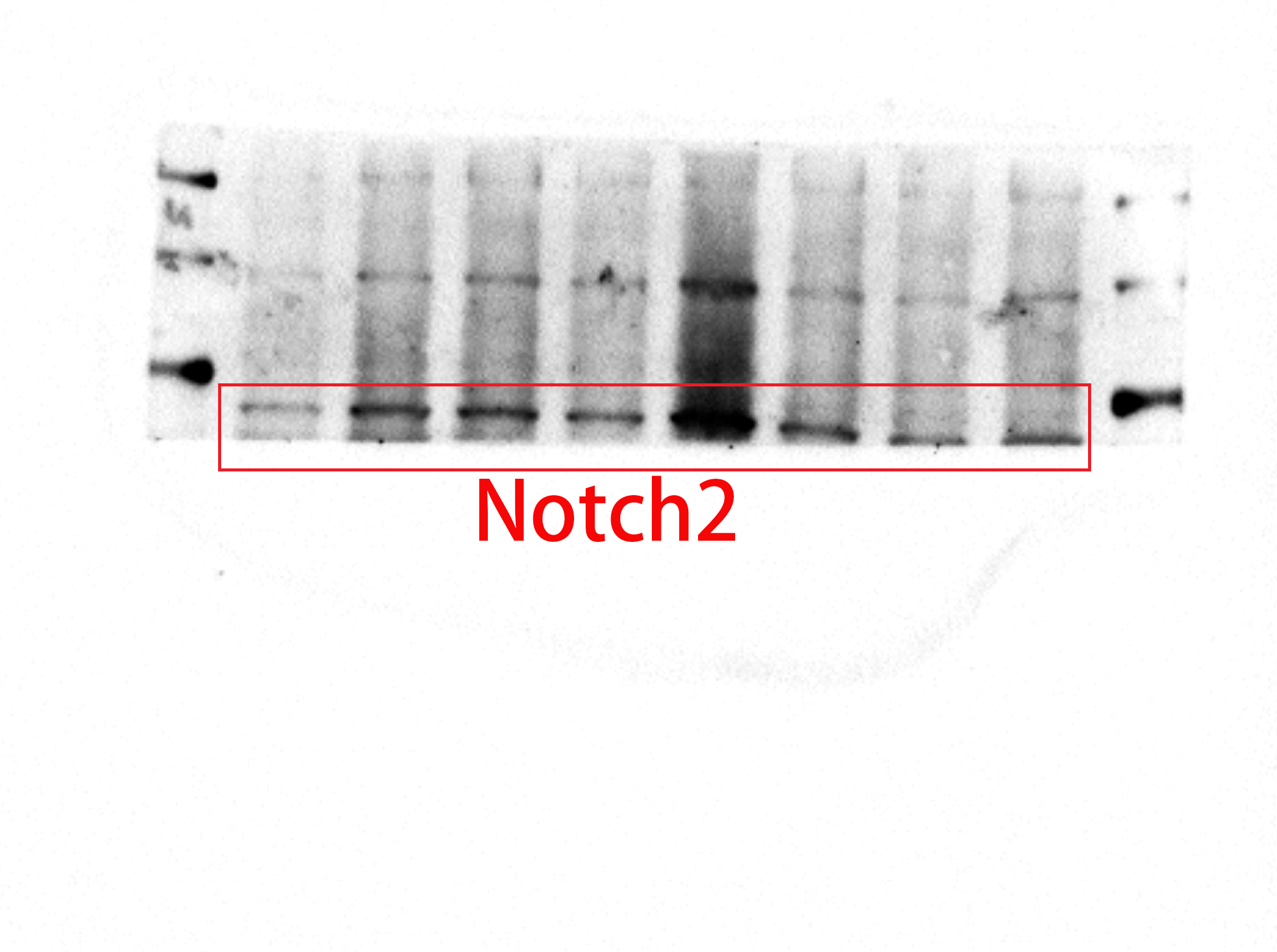

Supplement: Supplemental Information 3 [file peerj-09-11440-s003.zip › Uncropped GelsBlots/Figure7B.NOTCH2.tif]

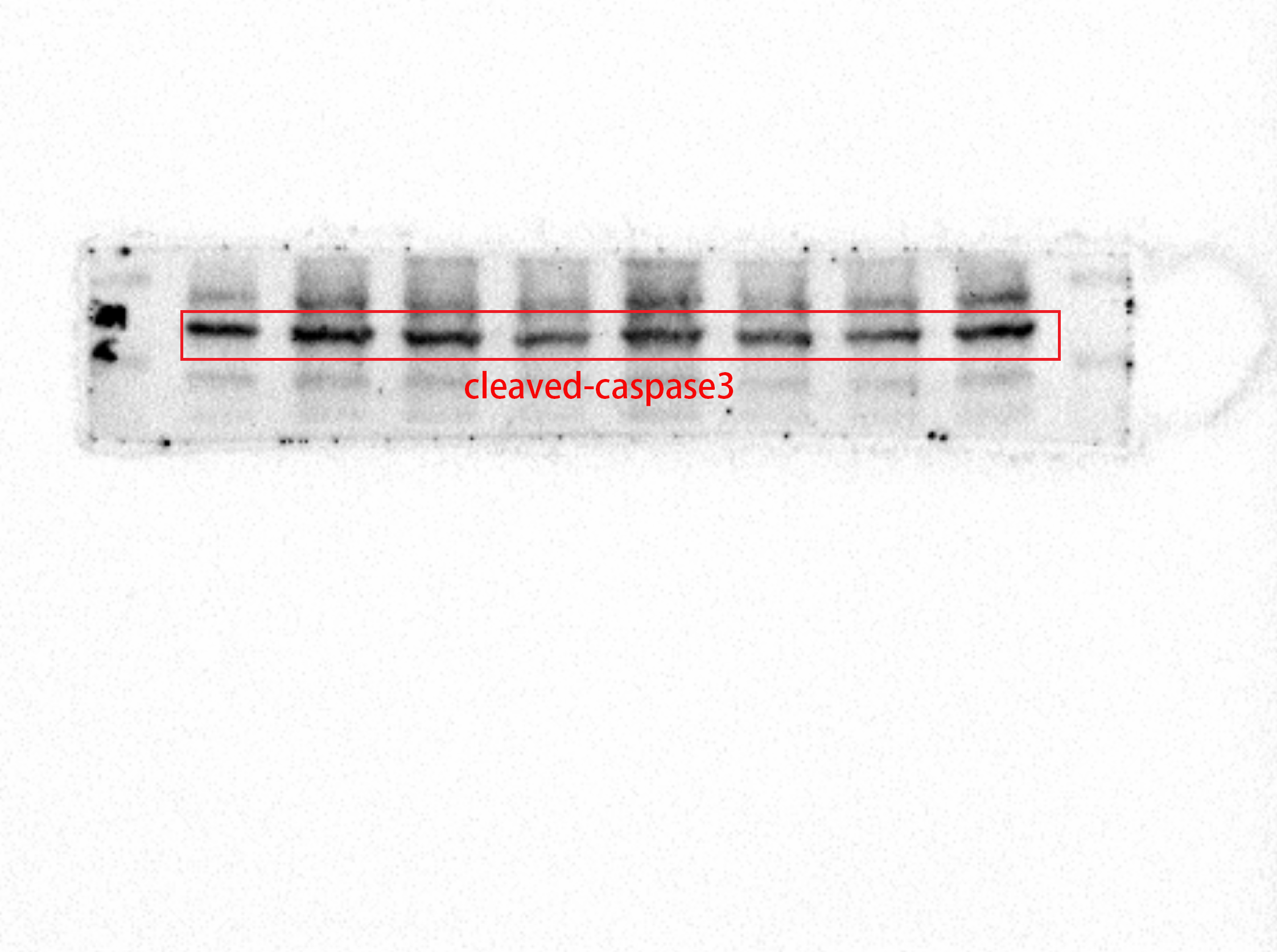

Supplement: Supplemental Information 3 [file peerj-09-11440-s003.zip › Uncropped GelsBlots/Figure7B.cleaved-caspase3.tif]

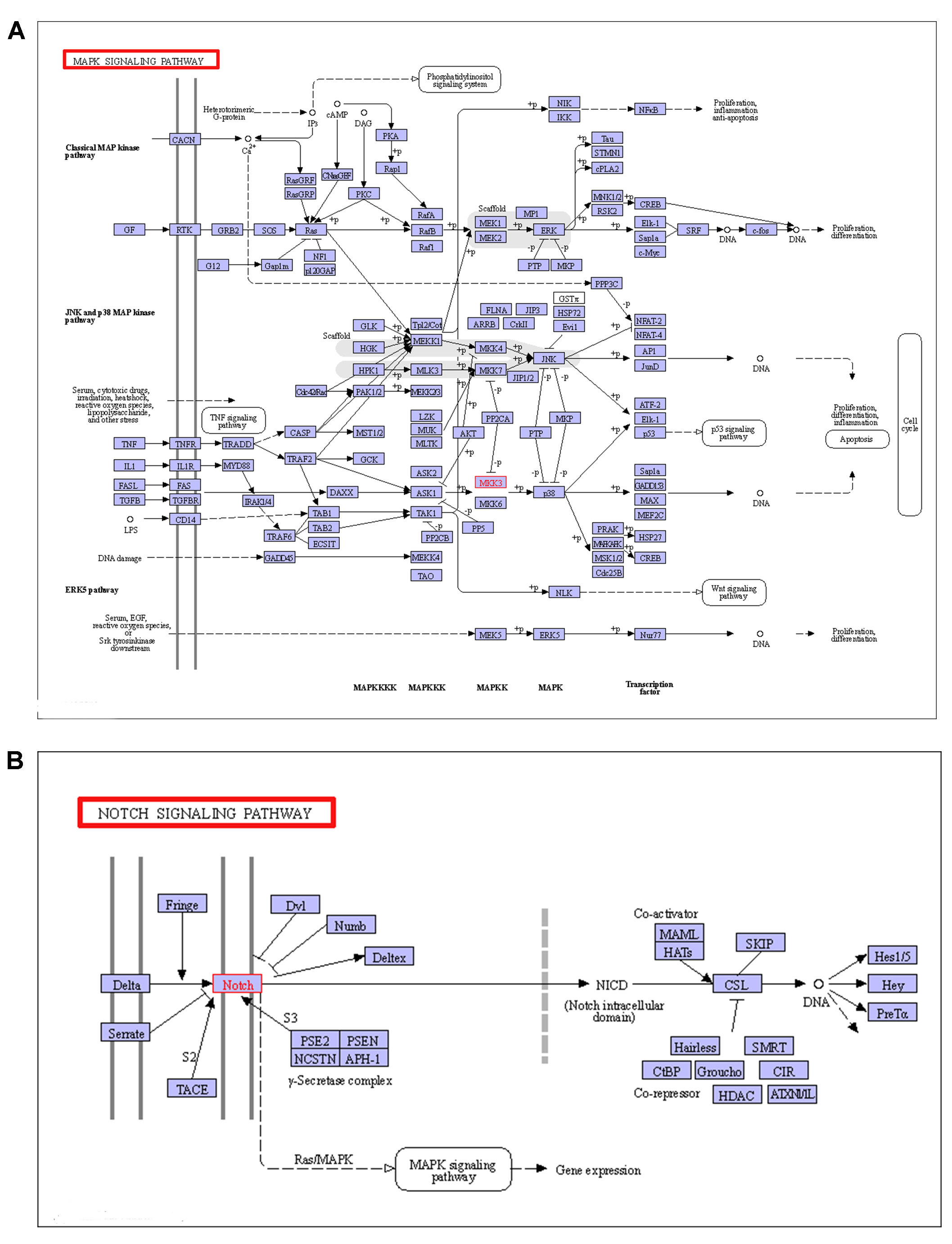

Supplement: Supplemental Information 4 [file peerj-09-11440-s004.tif]
